# The Diagnostic Value of Deep Learning for Multi-Classification of Rectal Cancer T Staging Based on Regional Attention

**DOI:** 10.3390/diagnostics16101525

**Published:** 2026-05-18

**Authors:** Chenyang Qiu, Yihui Xia, Zhiguo Feng, Kaige Liu, Rulei Zhong, Hongwu Liu, Hantao Zhang, Weidong Guo, Shouhong Wan, Wanqin Wang, Bingbing Zou

**Affiliations:** 1 Department of General Surgery, The First Affiliated Hospital of Anhui Medical University, Hefei 230022, China; 2 Institute of Artificial Intelligence, Hefei Comprehensive National Science Center, Hefei 230088, China; 3 The First Clinical Medical College, Anhui Medical University, Hefei 230032, China; 4 School of Computer Science and Technology, University of Science and Technology of China, Hefei 230026, China; 5 Department of Radiology, The First Affiliated Hospital of Anhui Medical University, Hefei 230022, China

**Keywords:** colorectal cancer, radiomics, deep learning, rectal cancer staging prediction model

## Abstract

**Objective**: To explore the feasibility and effectiveness of an enhanced CT deep learning model based on regional attention for the preoperative multi-classification of rectal cancer T stages. **Methods**: Five hundred eligible patients with rectal cancer (48 in T1 stage, 127 in T2, 259 in T3, and 66 in T4) were randomly divided into a training group (*n* = 400) and a validation group (*n* = 100). Regions of interest (ROIs) in rectal cancer lesions were pixel-wise annotated by experienced radiologists. A deep learning algorithm based on regional attention was used to train a binary classification model (early stage—T1 and T2, advanced stage—T3 and T4) and a multi-classification model (T1, T2, T3 and T4 stages), which were compared against radiomics approaches. Features were extracted from manually segmented ROIs using pyradiomics, radiomics-based binary and multi-classification models using ten different algorithms. In addition, baseline clinical data-based binary and multi-classification models were also constructed. The performance of both binary and multi-classification models were evaluated by plotting receiver operating characteristic (ROC) curves. The area under the curve (AUC) and accuracy were calculated for the binary model, and the micro-average AUC, macro-average AUC, and accuracy were calculated for the multi-classification model. **Results**: The ROI-based binary classification model for T stage (ROITransStage; AUC = 0.878, accuracy = 0.850) outperformed the best among ten radiomics-based binary models (AdaBoost; AUC = 0.802, accuracy = 0.76), as well as the best-performing baseline clinical data binary model (AdaBoost; AUC = 0.836, accuracy = 0.76). In addition, ROITransStage (micro-average AUC = 0.873, macro-average AUC = 0.862, accuracy = 0.81) also demonstrated superior diagnostic performance for the T1, T2, T3 and T4 stages compared to the best-performing radiomics-based (SVM; micro-average AUC = 0.845, macro-average AUC = 0.777, accuracy = 0.6) and baseline clinical data-based (SVM; micro-average AUC = 0.841, macro-average AUC = 0.76, accuracy = 0.61) multi-classification models. **Conclusions**: The CT deep learning binary and multi-classification models based on regional attention exhibited superior predictive performance for rectal cancer staging compared to both radiomics and clinical data-based models.

## 1. Introduction

Colorectal cancer ranks third in terms of incidence and second in terms of cancer-related mortality rate worldwide [1,2]. Rectal cancer accounts for one-third of all colorectal cancer cases, and is most prevalent in East Asia [3]. At present, the treatment of rectal cancer primarily involves surgery in combination with radiotherapy and chemotherapy. The combined approach has extended the disease-free survival period for rectal cancer patients, leading to longer expected lifespans and improved quality of life. In addition, neoadjuvant chemoradiotherapy based on preoperative staging has increased the R0 resection rate of tumors [4,5,6]. The optimum treatment plan for rectal cancer depends on accurate preoperative staging, and immediate surgical resection is not possible for all diagnosed patients. Furthermore, the specific surgical approach and the need for neoadjuvant chemoradiotherapy vary depending on the location, size and stage of the tumors, and the choice of treatment strategy significantly impacts the patient’s quality of life and prognosis [7]. Therefore, accurate preoperative T staging of rectal cancer is critical to select the best treatment strategy for patients.

Currently, the primary imaging modalities used for the preoperative staging of rectal cancer include endoscopic ultrasound (EUS), positron emission tomography–computed tomography (PET-CT), magnetic resonance imaging (MRI), and computed tomography (CT). Regarding loco-regional staging, each modality presents distinct advantages and limitations. EUS demonstrates high accuracy in evaluating the early involvement of the rectal wall layers; however, its invasiveness reduces patient acceptance, and it is inadequate for comprehensively assessing distant metastases. While PET-CT is highly valuable for whole-body evaluation, its high cost and associated radiation risks generally preclude its use as a routine modality for local staging. In contrast, high-resolution MRI, with its exceptional soft-tissue contrast, enables precise assessment of tumor invasion and is widely recognized as the undisputed gold standard for the local staging of rectal cancer. Nevertheless, MRI requires high patient compliance and entails long scanning and appointment waiting times. Furthermore, the limited availability of MRI equipment, particularly in primary care facilities, significantly restricts its universal applicability for routine staging. Consequently, contrast-enhanced CT, characterized by its non-invasive nature, rapid acquisition, and widespread accessibility, remains a broadly adopted routine staging modality for comprehensive whole-body assessment. Despite its extensive clinical use, CT possesses notable limitations in loco-regional staging; its inferior soft-tissue contrast resolution presents significant challenges for accurate local evaluation, such as T staging. Given the extensive clinical reliance on CT and its inherent limitations in local assessment, exploring novel auxiliary analytical techniques (such as deep learning) to overcome its staging deficiencies holds immense clinical significance.

Radiomics is defined as the extraction of biological and pathological features from medical imaging data, such as CT and MRI, in the form of quantitative metrics [8]. The extraction of morphological and textural features that are difficult to discern with the naked eye can aid in the selection of optimal treatment strategies for rectal cancer patients [9,10]. Prior studies have utilized radiomics to develop MRI-based models for predicting the preoperative T stage of rectal cancer [11]. Furthermore, while some research has applied deep learning approaches for the detection and segmentation of rectal tumors, the majority of these investigations have predominantly focused on MRI or PET imaging [12,13]. In contrast, deep learning studies utilizing routine contrast-enhanced CT (CECT) for the precise loco-regional staging of rectal cancer remain relatively scarce. Given the large patient population and the uneven distribution of medical resources, automated assessment of local invasion using CECT can significantly enhance diagnostic efficiency and provide an objective preoperative reference for clinicians. Therefore, this study specifically focuses on the primary rectal tumor, aiming to fill the literature gap regarding CECT-based deep convolutional models for loco-regional staging.

The aim of this study was to develop deep learning methods for preoperative prediction of rectal cancer T staging, and provide a non-invasive, simple, and economical option for clinical assessment.

## 2. Materials and Methods

### 2.1. Patient Selection

**Figure 1:**
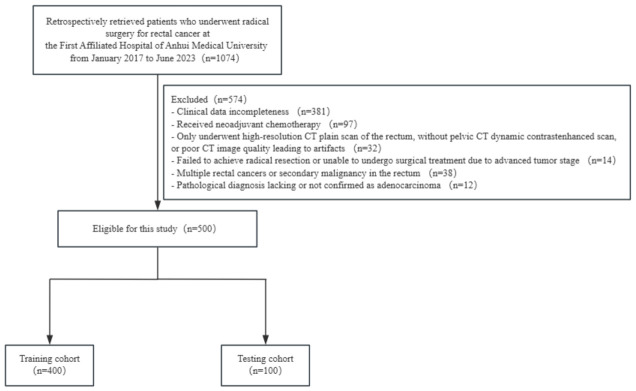
Flowchart of patient recruitment in this study.

A total of 500 rectal cancer patients who underwent radical surgery at the First Affiliated Hospital of Anhui Medical University from January 2017 to June 2023 were retrospectively analyzed. The inclusion criteria were as follows: (1) availability of complete clinical data, (2) no prior neoadjuvant chemotherapy, (3) availability of complete high-resolution CT scans of the rectum and contrast-enhanced CT scans in the portal venous phase, (4) successful tumor resection, (5) the presence of a solitary primary malignant tumor in the rectum, and (6) confirmed pathological diagnosis of adenocarcinoma. The exclusion criteria were as follows: (1) incomplete clinical data, (2) prior neoadjuvant chemotherapy, (3) only underwent high-resolution CT scans of the rectum without pelvic CT dynamic enhancement scanning, or the CT images were of poor quality with artifacts, (4) inability to achieve radical resection or inoperability due to advanced tumor staging, (5) presence of multiple rectal tumors or secondary tumors in the rectum, and (6) lack of pathological diagnosis or no confirmation of adenocarcinoma. The clinical data and the reports of laboratory tests and pathological diagnosis were retrieved from the electronic medical record system. The eligible patients were randomly divided into a training cohort (*n* = 400) and a validation cohort (*n* = 100) at a ratio of 4:1 (Figure 1).

### 2.2. Imaging

Contrast-enhanced CT scans from the abdomen to the pelvis were performed using a 64-slice CT scanner (Revolution CT; GE Medical Systems, Chicago, IL, USA). An individualized, weight-based dose of 1.5 mL/kg of iohexol (Omnipaque; GE Healthcare, Shanghai, China) was administered via the cubital vein. For image acquisition, a bolus tracking technique was employed. Specifically, the portal venous phase imaging was acquired with a precise diagnostic delay of 50 s after the contrast attenuation reached a predefined threshold of 100 HU within the abdominal aorta. Each CT scan was acquired using the following parameters: slice thickness—5 mm, reconstruction interval—0.625 mm, tube voltage—120 kVp, tube current—8 to 420 mA, high-resolution matrix size—512 × 512, field of view—500 mm, rotation time—0.75 s, pitch—0.984, and pixel size—1.46 mm.

**Figure 2:**
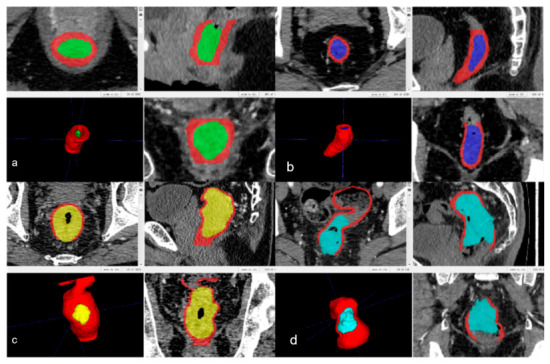
Pixel-wise annotation results of tumors at different T stages. (**a**–**d**) illustrate the pixel-level annotations of tumors at various stages, where red represents normal rectal wall structure, green indicates T1 stage tumor involvement, purple indicates T2 stage tumor involvement, yellow indicates T3 stage tumor involvement, and blue indicates T4 stage tumor involvement.

During the portal venous phase, the tumor lesions were significantly enhanced, allowing for easier differentiation between the tumor area and the surrounding normal tissues. Several studies have utilized portal venous phase enhanced CT images for tumor lesion segmentation [14]. The enhanced CT images of each patient were exported, and each slice was manually segmented using the ITK-SNAP software (version 3.8.0, www.itksnap.org). The tumor lesions on each slice were delineated pixel-wise along the tumor contours, while avoiding the intestinal contents and air. CT-based T staging was evaluated to best approximate the pathological categories of the 8th edition of the AJCC TNM classification. Since the AJCC manual does not explicitly provide detailed CT morphological standards, the specific imaging criteria utilized in our study were established based on the consensus clinical experience of specialized radiologists and gastrointestinal surgeons, alongside established morphological descriptions from relevant radiological literature [15,16]. The detailed CT evaluation criteria were as follows: (1) T1 stage tumors invade the submucosa but do not involve the muscularis propria, and are characterized by localized enhancement lesions within the submucosa, lesions appearing as well-defined soft-tissue density masses without thickening of the bowel wall, and smooth and clear outer edge of the bowel wall. (2) T2 stage tumors invade the muscularis propria and are confined to the bowel wall, without invasion of the external muscle layer. Localized enhancement lesions are observed in the rectal wall, and the tumors vary in size and often appear lobulated and asymmetric. When the long axis of the tumor aligns with the scanning plane, the bowel wall appears as tubular irregular thickening, stiffness, and narrowing of the lumen, and the outer edge of the bowel wall is smooth and clear. (3) T3 stage tumors grow through the muscularis propria into the surrounding mesorectum, and are characterized by a rough and unclear serosal surface of the bowel wall. Tumor invasion into the mesorectum manifests as an increase in needle-like density within the mesorectal fat, or deformation and distortion of the muscularis propria. Larger tumors may contain low-density ischemic necrosis areas. (4) T4 stage tumors invade the surrounding structures such as the peritoneal reflection, pelvic wall, vagina, prostate, bladder, or seminal vesicles, and are characterized by increased density, the presence of speckled, striated shadows, and soft-tissue shadows within the surrounding fat tissue, or absence of fat space between adjacent organs. Larger tumors may also contain low-density ischemic necrosis areas. The manually annotated enhanced CT images are shown in Figure 2.

Enhanced CT images of 40 randomly selected subjects, including 10 cases each of T1, T2, T3, and T4 stage tumors, were analyzed by two radiologists (Radiologist 1 and Radiologist 2) who were blinded to all clinical information except tumor location. The patients were staged based solely on the enhanced CT findings. The tumor conditions on each frame of the enhanced CT were annotated, and revisions were made on the sagittal and coronal planes after marking on the axial plane to ensure the uniformity of the final ROI annotations across axial, sagittal, and coronal views. The inter-observer variability of the radiomics features was calculated based on the independent annotations by the two radiologists, and the intra-observer variability was calculated based on the two sets of annotations performed one month apart by Radiologist 1. The intraclass correlation coefficient (ICC) was calculated, and ICC > 0.75 was indicative of good reproducibility. Radiologist 1 completed the marking of the remaining subjects and reconstructed the volume of interest (VOI) based on the ROI of each patient. A total of 500 representative preoperative enhanced CT cases were selected, and 34,364 out of 376,605 frames were annotated for model training.

### 2.3. Data Preprocessing

**Figure 3:**
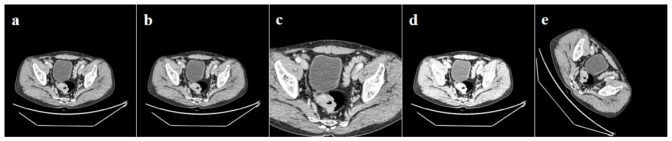
Preprocessing of CT slices. (**a**), original image; (**b**), normalization; (**c**), cropping and resizing; (**d**), contrast adjustment; (**e**), rotation.

Before model training, the annotated enhanced CT images were segmented into slices according to the annotated frames, and the format of the slices was confirmed to be correct. The enhanced CT slices were adjusted based on empirically derived window width (400) and level (75), and the Hounsfield Unit (HU) values were constrained within (−125, 275) and normalized to (0, 1). The mean and standard deviation of all slices in the training set were calculated for normalization. The CT slices underwent contrast adjustment, rotation, cropping, and resizing to ensure the consistency and processability of the image data (Figure 3). The 2D CT images (data processing object is a single-frame slice) were directly used for training, whereas the 3D CT image slices (data processing object is the annotated complete tumor) underwent sampling reorganization before training.

### 2.4. Construction of Deep Learning Model Based on Regional Attention

The hardware and software configuration for the deep learning model were as follows: AMD EPYC 7742 64-core 3.4 GHz CPU, 256 GB memory, NVIDA GeForce RTX3090 GPU with 24 GB of video memory, Ubuntu 20.04 operating system, PyTorch1.10.1 deep learning framework, and Python 3.9 code. The distribution of the overall sample was assessed, and kept consistent when splitting the dataset to ensure the model’s generalization ability. The ratio of the training set to the validation set was 4:1 (400 samples and 100 samples).

**Figure 4:**
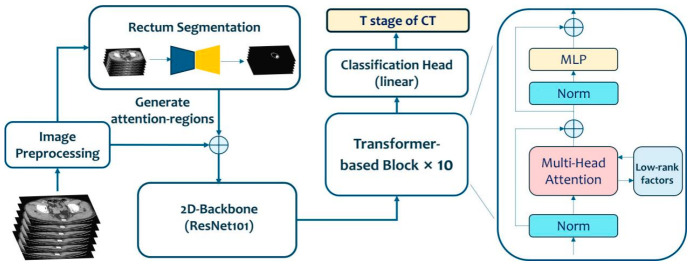
Architecture of the deep learning network.

As shown in Figure 4, the ROITransStage model initially segmented the enhanced CT images into two-dimensional transverse slices, and enhanced the contrast and normalization in each frame slice. The preprocessed slices are fed into the Segment Anything Model (SAM) [17], which segmented the rectum and rectal tumor area from the enhanced CT images and generated features corresponding to the original CT frames. These segmented areas are used to locate the rectal tumor and seamlessly integrate the features of the tumor area into the original CT frames. The feature-integrated images were then input into ResNet101 for feature extraction, and the extracted feature sequences were fine-tuned in a Transformer block pre-trained on the ImageNet dataset. The Transformer block includes a Layer Normalization (LN) layer, a multi-head attention mechanism, and residual connections composed of LN, an MLP block, Dropout, etc. The MLP block consists of a linear layer, an activation function, and Dropout. After processing through the Transformer block, the T stage of the enhanced CT images was determined using a linear classification head. During the training phase, the weights of the Transformer block were frozen, and a low-rank fine-tuning strategy was adopted by adding low-rank factors. A large number of parameters were converted to sufficient but non-redundant parameters through low-rank decomposition, thereby facilitating the transfer of data. In the testing phase, the weights of the backbone network completed the inference along with the low-rank parameters. During training, cross-entropy loss was used to stage the 2D CT slice images. Given that each case is a 3D CT image, a sequence testing task was adopted in the validation set. The tumor frame sequence of the original CT image was obtained through the rectal segmentation network and input into the network, and the staging information of all frames were integrated to determine the T stage.

In addition to ROITransStage, two commonly used deep learning network frameworks, Convnext [18] and Vision Transformer [19], were also trained. The experimental results of the three models were compared.

### 2.5. Feature Selection and Radiomics Model Construction

**Figure 5:**
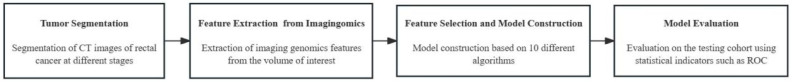
Workflow of radiomics model construction.

The radiomic features were initially normalized using the Z-score to transform the data to follow a normal distribution N~(0, 1), and then screened through a *t*-test (*p* < 0.05 was considered significant). For the features exhibiting high repeatability, Pearson’s correlation coefficient was calculated to determine the inter-feature correlations, and one of any two highly correlated features (Pearson correlation coefficient > 0.9) were retained. The Least Absolute Shrinkage and Selection Operator (LASSO) algorithm was then used on the 12 retained features for further dimensionality reduction and optimization, and 1 first-order feature, 5 shape features, and 6 higher-order texture features were selected to construct the radiomics model. The details of feature extraction are included in the Appendix A. The same training and validation sets as those used for the ROITransStage model were adopted, and task learning was conducted using ten different machine learning algorithms, resulting in AdaBoost, ExtraTrees, GradientBoosting, KNN, LightGBM, Model MLP, NaiveBayes, RandomForest, SVM, and XGBoost models. The predictive performance of the models was evaluated by plotting receiver operating characteristic (ROC) curves, and calculating the micro-average area under the curve (AUC), macro-average AUC, and accuracy (Figure 5).

### 2.6. Construction of the Clinical Baseline Data Model

To eliminate the influence of unnecessary factors, the construction process of the clinical data model was almost identical to that of the radiomics model. Six clinical variables (including N stage, tumor length, PNI, LVI, CA199, and gender) were screened based on the Pearson’s correlation coefficient, and then optimized using the LASSO regression algorithm. The training and validation sets were the same as that used for ROITransStage, and the same machine learning algorithms used in the radiomics model were employed for task learning. Subsequently, the most effective algorithm was chosen to construct the model. The predictive performance of the clinical data-based model was evaluated with the metrics used for the radiomics model.

### 2.7. Radiomics Feature Processing and Model Development

A total of 107 radiomics features were extracted from each enhanced CT sequence, including 18 first-order features, 14 shape features, and 75 higher-order texture features. Of these 107 features, 12 were retained based on their Pearson correlation coefficients with tumor T staging. Finally, 1 first-order feature, 5 shape features, and 6 higher-order texture features were selected for constructing the radiomics model.

## 3. Results

### 3.1. Sample Details

**Figure 6:**
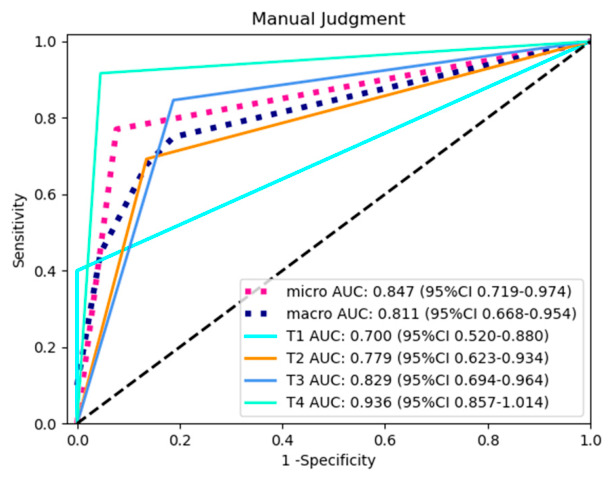
ROC curve of radiologists’ assessment of rectal cancer on contrast-enhanced CT.

A total of 500 patients, comprising 324 males and 176 females, with ages ranging from 31 to 90 years, were included in this study. There were 133, 300 and 67 cases of upper, middle, and lower rectal tumors respectively. According to the 8th edition of the TNM classification by the American Joint Committee on Cancer, 48 patients were classified as TNM stage T1, 127 as stage T2, 259 as stage T3, and 66 as stage T4. Tumor T staging, N staging, grade, and length are summarized in Table 1. The accuracy of T stage prediction based on enhanced CT images without pathological information was 0.77, and the respective accuracies for T1, T2, T3, and T4 stage tumors were 0.6, 0.692, 0.846, and 0.916. The ROC curves are shown in Figure 6.

### 3.2. Predictive Performance of Deep Learning Model for T Staging

**Figure 7:**
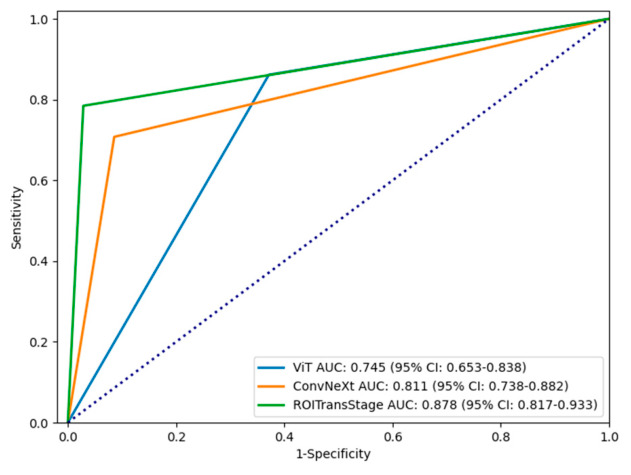
ROC curve of the deep learning binary classification model.

**Figure 8:**
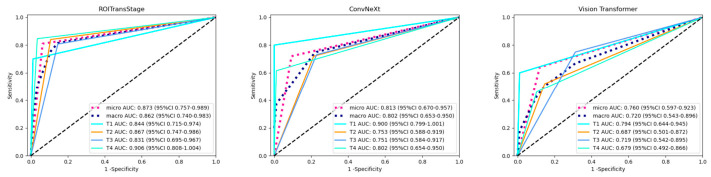
ROC curve of the deep learning multi-class classification model.

**Figure A1:**
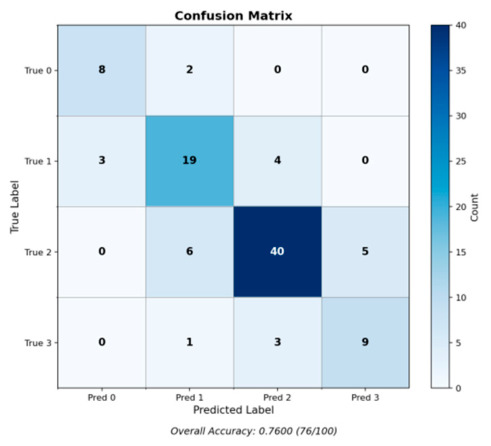
Confusion matrix of the ROITransStage multi-classification model.

**Figure A2:**
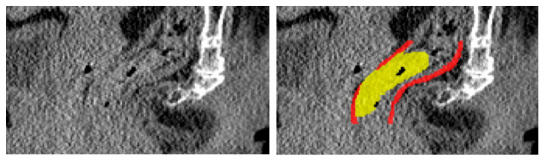
A T3 stage tumor misclassified as T2 by the model.

**Figure A3:**
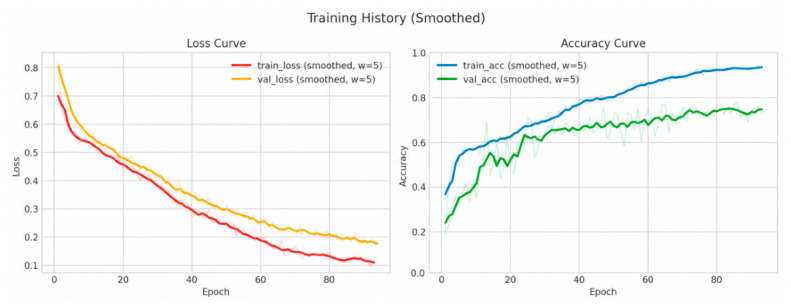
Loss function and accuracy curves during model training.

**Figure A4:**
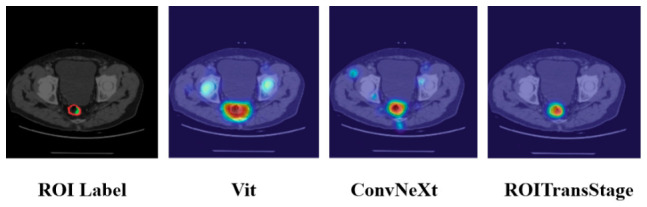
Grad-CAM visualization results of different models.Warmer colors (e.g., red and yellow) indicate regions receiving higher attention weights and contributing more strongly to the model’s decision, whereas cooler colors (e.g., blue and green) indicate regions with lower attention weights and less contribution.

The predictive performance of the ROITransStage model for the binary classification was evaluated in the validation set. As shown in Figure 7, the AUC and accuracy of ROITransStage were 0.878 and 0.850, respectively. Based on the ROC metrics, the predictive performance of the ROITransStage surpassed that of Convnext (AUC = 0.753, accuracy = 0.780) and Vision Transformer (AUC = 0.745, accuracy = 0.750) models. For the multi-classification approach, we evaluated both micro-average AUC (which aggregates the contributions globally to reflect overall performance) and macro-average AUC (which treats all classes equally to adjust for potential class imbalance). For the multi-classification approach, the micro-average AUC and macro-average AUC of the ROITransStage model were 0.873 and 0.862, respectively, the accuracy was 0.81 (Figure 8), and the metrics were superior to that of the Convnext (micro-average AUC = 0.813, macro-average AUC = 0.802, accuracy = 0.71) and Vision Transformer (micro-average AUC = 0.760, macro-average AUC = 0.72, accuracy = 0.63) models. To address class imbalance and comprehensively evaluate per-category performance, macro-average metrics were calculated, showing stable discrimination across all T stages (Appendix A Table A1). Furthermore, a confusion matrix was generated to detail the classification behavior, which revealed that misclassifications primarily occurred between adjacent stages rather than as severe cross-stage errors (Appendix A Figure A1). A clinical evaluation of the misclassified cases was also conducted. A representative case of a T3 tumor incorrectly downstaged to T2 by the model is analyzed in Appendix A Figure A2, highlighting the inherent limitations of CT soft-tissue contrast in detecting micro-invasion. The proposed model demonstrated stable convergence and good generalization during the training process, without evidence of severe overfitting (see Appendix A Figure A3 for detailed training loss and accuracy curves). Finally, Grad-CAM visualizations were utilized to assess the interpretability of the model. The results confirmed that the introduced regional attention mechanism effectively guided the model to focus on clinically relevant lesion areas, matching the diagnostic logic of medical experts (Appendix A Figure A4).

### 3.3. Predictive Performance of the Radiomics Model

**Figure 9:**
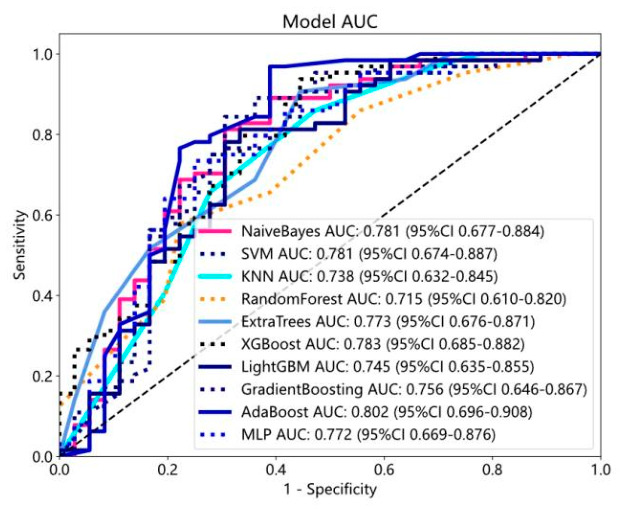
ROC curve of the radiomics binary classification model.

**Figure 10:**
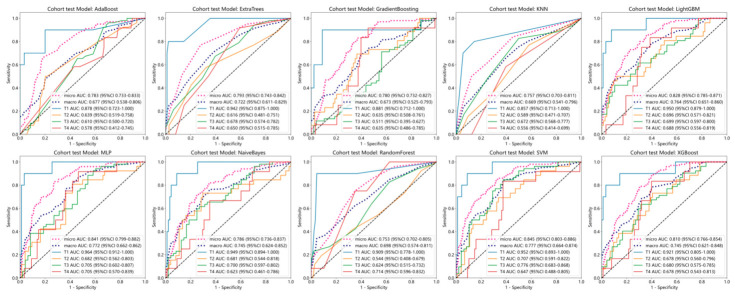
ROC curve of the radiomics multi-class classification model.

The ROC metrics of the radiomics model for binary classification in the training set are shown in Figure 9. The micro-average AUC and macro-average AUC of AdaBoost, ExtraTrees, GradientBoosting, KNN, LightGBM, MLP, NaiveBayes, RandomForest, SVM, and XGBoost multi-classification models in the test set are provided in Table 2 and ROC curves are in Figure 10. The results indicate superior predictive performance of the SVM model compared to the other radiomics models.

### 3.4. Predictive Performance of the Baseline Clinical Data Model

**Figure 11:**
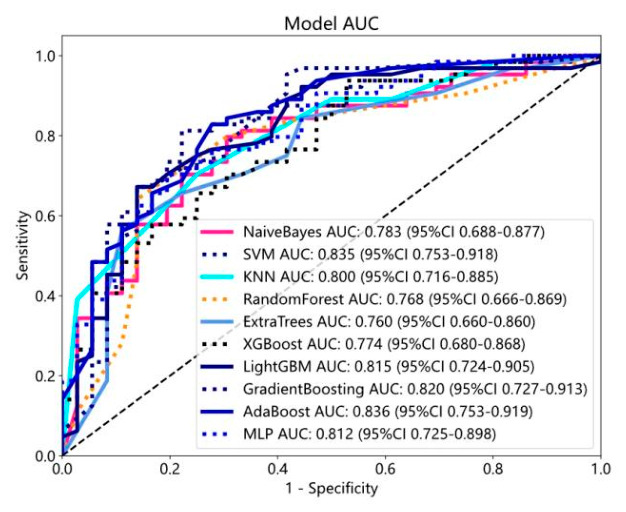
ROC curve of the clinical baseline binary classification model.

**Figure 12:**
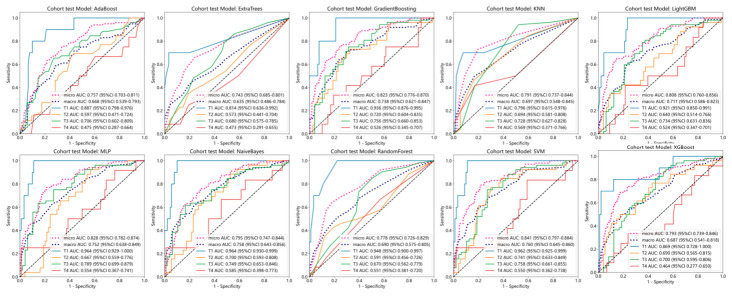
ROC curve of the clinical baseline multi-class classification model.

The ROC metrics of the clinical data-based binary classification model are shown in Figure 11. The baseline clinical data used for constructing the multi-classification model include N staging, tumor length, PNI, LVI, CA199, and gender. The micro-average AUC and macro-average AUC of the AdaBoost, ExtraTrees, GradientBoosting, KNN, LightGBM, MLP, NaiveBayes, RandomForest, SVM, and XGBoost models in the training and validation sets are shown in Table 3, the accuracies are summarized in Table 4, and the ROC curves are shown in Figure 12. According to these metrics, the predictive performance of the SVM clinical data model surpassed that of the other models.

Among all 23 multi-classification diagnostic models, the deep learning model ROITransStage exhibited a macro-average AUC of 0.862, outperforming all other models. This indicates that, overall, the ROITransStage has relatively good classification performance across all categories without any significant weaknesses. Additionally, the micro-average AUC of the ROITransStage model was 0.873, which surpassed that of other models, indicating relatively good classification performance of the deep learning model in each specific category. ROITransStage also showed the highest accuracy of 0.81 in the multi-classification diagnosis for rectal cancer T staging among the 23 models.

## 4. Discussion

The overall predictive capability of our deep learning model for rectal cancer T staging was superior to that of the radiomics-based model. Radiologists often misclassify T1 stage tumors as T2, and T2 stage tumors as T3 when relying solely on CT images [20]. A fundamental challenge in segmenting rectal tumors is that standard CT lacks sufficient soft-tissue resolution to reliably distinguish the distinct histological layers of the rectal wall. Faced with such blurred anatomical boundaries, radiologists tend to err on the side of caution to avoid under-staging and the risk of further development. To overcome these inherent visual limitations, the deep learning model based on regional attention and trained with a large data volume can eliminate the influence of subjective factors, thus allowing accurate identification of the subtle differences between various tumor stages, and enhancing the differentiation between T1 and T2 stage tumors.

Previous studies on rectal cancer diagnostics have mainly employed binary classification models, which are preferred in clinical practice due to their ease of operation and interpretation. For instance, Hou et al. [21] classified T1 and T2 stage rectal tumors as early stage, and T3 and T4 stage tumors as advanced stage using high-resolution MRI scans (HRT2) and super-resolution MRI data (SRT2) with deep learning techniques. The AUC and accuracy of the HRT2 model were 0.81 and 0.773, respectively, and those of the SRT2 model were 0.869 and 0.833. We constructed both binary and multi-classification models to categorize the rectal tumors as early or advanced, and further divide them into the T1, T2, T3 and T4 stages to facilitate clinical decision-making. The treatment approach for rectal cancer depends on the tumor stage. For instance, transanal local excision or transrectal endoscopic surgery are considered for T1 stage rectal tumors. These surgeries are minimally invasive with quicker recovery times, although it is crucial to ensure that the deep and mucosal margins of the excised specimen are negative for tumor cells. In the case of unfavorable features like positive margins, lymphovascular invasion, low differentiation, or invasion into the lower third of the submucosa, more extensive resection is recommended. Currently, local excision after neoadjuvant chemoradiotherapy may be considered for T2 stage rectal cancer in patients who refuse or are unfit for abdominal surgical resection. For T3 stage rectal cancer, the optimum treatment approach is surgery after neoadjuvant chemoradiotherapy, adjuvant chemotherapy before or after surgery, and possible targeted therapy. The surgical resection methods include abdominal resection or rectectomy. For T4 stage rectal cancer patients who cannot undergo surgical resection, palliative radiotherapy or chemotherapy may be considered to alleviate symptoms and control disease progression [22]. Therefore, a multi-classification approach can more accurately guide treatment decisions for rectal cancer patients, and improve patients’ quality of life and survival rates. Moreover, our binary classification model based on regional attention and trained with enhanced CT images demonstrated highly competitive diagnostic efficacy, achieving results comparable to previously reported models trained on high-resolution MRI data. While MRI remains the clinical gold standard, this finding highlights the potential of our AI-assisted CECT approach as a robust and reliable alternative when MRI is contraindicated or unavailable. The ROC metrics of the multi-classification model were slightly lower compared to that of the binary diagnostic model, possibly due to the increased difficulty in further subdividing early and advanced tumors into the T1, T2, T3, and T4 stages. Furthermore, the AUC for distinguishing T2 from T1, T3, and T4 stages was 0.773, and for T3 from T1, T2, and T4 stages, it was 0.739, indicating that ROITransStage has relatively lower accuracy in differentiating between T2 and T3 stage rectal cancer. This might be due to similarities in imaging features between the T2 and T3 stage tumors. T2 stage tumors show relatively uniform density and lower enhancement compared to the surrounding normal rectal wall, with no significant abnormal enhancement around the vessels. On the other hand, T3 stage tumors often exhibit a more heterogeneous enhancement pattern, possibly with signs of central necrosis or hemorrhage, and the surrounding vessels may show abnormal enhancement that corresponds to changes in blood supply. However, T2 stage tumors that reach the thickness of the deep muscle layer and T3a stage tumors that invade > 1 mm into the muscularis propria have similar imaging characteristics [23]. Given the relatively limited soft-tissue contrast resolution of CT scans, it may be difficult to accurately detect these subtle changes in tumor tissue structure, and differentiate between the two stages.

Due to the complex tissue structure of the rectal area, it is challenging for convolutional neural networks to distinguish between tumor and normal rectal tissues. However, deep learning methods based on regional attention can more precisely locate and identify tumor sites within the rectal area [24,25]. Therefore, we adopted this deep learning model for processing enhanced CT data of rectal cancer. The deep learning network was provided with granular, pixel-level annotations of rectal CT images. Through pre-training, the network learned to perceive the rectal cancer area, focusing more attention on the tumor region when processing the enhanced CT data. This method significantly improved the accuracy and reliability of tumor detection, especially for early and localized rectal cancers, which can translate to more precise diagnosis and treatment of rectal cancer.

Studies similar to ours had adopted a bounding box drawing method for labeling samples, and marked tumor areas with rectangular boxes to obtain the ROI for training deep learning models [26]. This method is simple, efficient, and suitable for tumors of various sizes and shapes. However, tumors often have complex shapes and edges, and using a single rectangular boundary box may not capture their details and accurate boundaries, nor fully analyze and model three-dimensional information. Therefore, we used a pixel-level annotation method to ensure that each sample accurately reflects the inherent characteristics of tumor tissue and clearly depicts the staging of the tumor. Instead of marking indiscriminately, we performed initial annotations on the axial plane, and revised them on the sagittal and coronal planes to ensure the consistency, integrity, and coherence of the annotations. Pixel-level annotation better captures the details and boundaries of the tumor, which facilitates modeling of the three-dimensional information, thereby enhancing the quality of data and improving the performance of deep learning models. Moreover, the pixel-level annotation method helps create more representative training data, which can improve the generalization ability of the model. Thus, the model may achieve better predictive accuracy in new clinical scenarios, such as cases involving different individuals or rectal tumors of various shapes and sizes. Detailed annotation also enhances the interpretability of the model, which can aid in clinical decision-making under various circumstances. Although integrating clinical baseline data can enhance the performance of models for other diseases [27,28], some studies have reported that the addition of clinical data decreased the stability of deep learning models [29]. Given that the performance of our deep learning model was superior to that of traditional radiomics and clinical models, we did not integrate clinical data in order to increase its stability.

Manual pixel drawing increases the segmentation workload, thus limiting the expansion of the sample size. Utilizing automatic segmentation algorithms can reduce the workload and enhance accuracy, expand the volume of annotated image data, and improve the performance of rectal cancer diagnostic models. Furthermore, our findings are based on data from a single center, and require external validation. The performance and generalizability of the model across different datasets can be verified by applying it to multi-center data.

This study offers new insights into the precise application of AI in rectal cancer diagnosis. Rectal cancer is prone to misdiagnosis and missed diagnosis with traditional methods. AI, through deep learning algorithms, can automatically analyze medical images, providing more accurate, reliable, and timely diagnosis. We propose the specialized application of AI in precision medicine, enhancing treatment effectiveness, and reducing complications and recurrence rates.

## Figures and Tables

**Table 1:** Baseline clinical characteristics of the 500 patients.

	Total	Training Cohort (80%)	Testing Cohort (20%)	*p*
Gender				0.455
Male	324	256	68	
Female	176	144	32	
Age (mean ± SD, years)	62.95 ± 11.367	63.24 ± 11.224	61.78 ± 11.908	0.251
Tumor Size (mean ± SD, cm)	5.72 ± 2.928	5.77 ± 3.118	5.52 ± 1.995	0.453
Tumor Location				0.266
Low	171	134	37	
Medium	260	207	53	
High	69	59	10	
Pilnerval Intraepithelial Neoplasia				0.466
Negative	301	244	57	
Positive	199	156	43	
Lymphatic Invasion				0.780
Negative	321	258	63	
Positive	179	142	37	
T Stage				0.723
T1	48	38	10	
T2	124	99	25	
T3	262	210	52	
T4	66	53	13	
N Stage				0.139
N0	257	199	58	
N1	150	123	27	
N2	93	78	15	
Height (mean ± SD, cm)	165.51 ± 6.844	165.38 ± 6.853	166.02 ± 6.818	0.403
Weight (mean ± SD, kg)	62.10 ± 10.035	61.84 ± 9.996	63.16 ± 10.174	0.240
BMI (mean ± SD, kg/m^2^)	22.63 ± 3.148	22.56 ± 3.078	22.90 ± 3.415	0.329
Alpha Fetoprotein (AFP)				0.806
Negative(<7 ng/mL)	484	387	97	
Positive (>7 ng/mL)	16	13	3	
Carcinoembryonic Antigen (CEA)				0.696
Negative(<5 ng/mL)	353	281	72	
Positive (>5 ng/mL)	147	119	28	
CA 125				0.417
Negative(<35 U/mL)	486	390	96	
Positive (>35 U/mL)	14	10	4	
CA 19-9				0.534
Negative(<35 U/mL)	455	363	92	
Positive (>35 U/mL)	45	37	8	

**Table 2:** Micro-average AUC, macro-average AUC, and one-vs-rest AUC for each T stage of different radiomics multi-class classification models in the test set.

	Model AdaBoost	Model ExtraTrees	Model GradientBoosting	Model KNN	Model LightGBM	Model MLP	Model NaiveBayes	Model RandomForest	Model SVM	Model XGBoost
micro-average AUC	0.783 (95% CI 0.733–0.833)	0.793 (95% CI 0.743–0.842)	0.780 (95% CI 0.732–0.827)	0.757 (95% CI 0.703–0.811)	0.828 (95% CI 0.785–0.871)	0.841 (95% CI 0.799–0.882)	0.786 (95% CI 0.736–0.837)	0.753 (95% CI 0.702–0.805)	0.845 (95% CI 0.803–0.886)	0.810 (95% CI 0.766–0.854)
macro-average AUC	0.677 (95% CI 0.538–0.806)	0.722 (95% CI 0.611–0.829)	0.673 (95% CI 0.525–0.793)	0.669 (95% CI 0.541–0.796)	0.764 (95% CI 0.651–0.860)	0.772 (95% CI 0.662–0.862)	0.745 (95% CI 0.624–0.852)	0.698 (95% CI 0.574–0.811)	0.777 (95% CI 0.664–0.874)	0.752 (95% CI 0.638–0.849)
T1 AUC	0.878 (95% CI 0.723–1.000)	0.942 (95% CI 0.875–1.000)	0.881 (95% CI 0.712–1.000)	0.857 (95% CI 0.713–0.100)	0.950 (95% CI 0.879–1.000)	0.964 (95% CI 0.912–1.000)	0.949 (95% CI 0.894–1.000)	0.909 (95% CI 0.778–1.000)	0.707 (95% CI 0.664–0.874)	0.964 (95% CI 929–1.000)
T2 AUC	0.639 (95% CI 0.519–0.758)	0.616 (95% CI 0.481–0.751)	0.635 (95% CI 0.508–0.761)	0.589 (95% CI 0.471–0.707)	0.696 (95% CI 0.571–0.821)	0.682 (95% CI 0.562–0.803)	0.681 (95% CI 0.544–0.818)	0.544 (95% CI 0.408–0.679)	0.952 (95% CI 0.893–1.000)	0.667 (95% CI 0.559–0.776)
T3 AUC	0.610 (95% CI 0.5000–0.720)	0.678 (95% CI 0.574–0.782)	0.511 (95% CI 0.395–0.627)	0.672 (95% CI 0.568–0.777)	0.699 (95% CI 0.597–0.800)	0.705 (95% CI 0.602–0.807)	0.700 (95% CI 0.597–0.802)	0.624 (95% CI 0.515–0.732)	0.776 (95% CI 0.683–0.868)	0.789 (95% CI 0.699–0.879)
T4 AUC	0.578 (95% CI 0.412–0.745)	0.650 (95% CI 0.515–0.785)	0.635 (95% CI 0.486–0.785)	0.556 (95% CI 0.414–0.699)	0.688 (95% CI 0.556–0.819)	0.705 (95% CI 0.570–0.839)	0.623 (95% CI 0.461–0.786)	0.714 (95% CI 0.596–0.832)	0.647 (95% CI 0.488–0.805)	0.554 (95% CI 0.367–0.741)

**Table 3:** Micro-average AUC, macro-average AUC, and one-vs-rest AUC for each T stage of different clinical baseline models in the test set.

	Model: AdaBoost	Model: ExtraTrees	Model: GradientBoosting	Model: KNN	Model: LightGBM	Model: MLP	Model: NaiveBayes	Model: RandomForest	Model: SVM	Model: XGBoost
micro-average AUC	0.757 (95% CI 0.703–0.811)	0.743 (95% CI 0.685–0.801)	0.823 (95% CI 0.776–0.870)	0.791 (95% CI 0.737–0.844)	0.808 (95% CI 0.760–0.856)	0.828 (95% CI 0.782–0.784)	0.795 (95% CI 0.747–0.844)	0.778 (95% CI 0.726–0.829)	0.841 (95% CI 0.791–0.884)	0.793 (95% CI 0.739–0.846)
macro-average AUC	0.668 (95% CI 0.539–0.793)	0.635 (95% CI 0.486–0.784)	0.738 (95% CI 0.621–0.847)	0.697 (95% CI 0.548–0.845)	0.711 (95% CI 0.586–0.823)	0.752 (95% CI 0.638–0.849)	0.758 (95% CI 0.643–0.856)	0.690 (95% CI 0.575–0.805)	0.760 (95% CI 0.645–0.860)	0.687 (95% CI 0.541–0.818)
T1 AUC	0.887 (95% CI 0.798–0.976)	0.814 (95% CI 0.636–0.992)	0.936 (95% CI 0.876–0.995)	0.796 (95% CI 0.615–0.915)	0.921 (95% CI 0.850–0.991)	0.964 (95% CI 0.929–1.000)	0.964 (95% CI 0.930–0.999)	0.948 (95% CI 0.900–0.997)	0.962 (95% CI 0.925–0.999)	0.869 (95% CI 0.728–1.000)
T2 AUC	0.597 (95% CI 0.471–0.724)	0.573 (95% CI 0.441–0.704)	0.720 (95% CI 0.604–0.835)	0.694 (95% CI 0.581–0.808)	0.640 (95% CI 0.514–0.766)	0.667 (95% CI 0.559–0.776)	0.700 (95% CI 0.593–0.808)	0.591 (95% CI 0.456–0.726)	0.741 (95% CI 0.633–0.849)	0.690 (95% CI 0.565–0.815)
T3 AUC	0.706 (95% CI 0.602–0.809)	0.680 (95% CI 0.575–0.785)	0.756 (95% CI 0.660–0.853)	0.728 (95% CI 0.627–0.828)	0.734 (95% CI 0.631–0.836)	0.789 (95% CI 0.699–0.879)	0.749 (95% CI 0.653–0.846)	0.670 (95% CI 0.562–0.779)	0.758 (95% CI 0.661–0.855)	0.700 (95% CI 0.595–0.806)
T4 AUC	0.475 (95% CI 0.287–0.664)	0.473 (95% CI 0.291–0.655)	0.526 (95% CI 0.345–0.707)	0.569 (95% CI 0.371–0.766)	0.524 (95% CI 0.347–0.701)	0.554 (95% CI 0.367–0.741)	0.585 (95% CI 0.398–0.773)	0.551 (95% CI 0.381–0.720)	0.550 (95% CI 0.362–0.738)	0.464 (95% CI 0.277–0.650)

**Table 4:** Accuracy of radiomics multi-class classification models and clinical baseline multi-class classification models.

	Model AdaBoost	Model ExtraTrees	Model GradientBoosting	Model KNN	Model LightGBM	Model MLP	Model NaiveBayes	Model RandomForest	Model SVM	Model XGBoost
Radiomics multi-class classification models	0.620	0.550	0.510	0.570	0.590	0.620	0.490	0.470	0.600	0.530
Clinical baseline multi-class classification models	0.530	0.530	0.610	0.620	0.620	0.570	0.580	0.580	0.610	0.590

## Data Availability

The original contributions presented in this study are included in the article. Further inquiries can be directed to the corresponding authors.

## Appendix A

### Appendix A.1

To further analyze the model’s classification behavior, we generated a confusion matrix (Figure A1). The sample distribution for each category is primarily concentrated along the diagonal. This shows that the model maintains good discrimination ability across different lesions, despite a certain degree of class imbalance. Misclassifications mainly occur between adjacent categories (e.g., T2 and T3), with rare instances of severe, multi-stage misjudgments. This performance is consistent with the clinical reality that adjacent-stage lesions often share similar features and have indistinct boundaries. Also, by avoiding severe cross-stage errors, the model demonstrates its safety and potential value in clinical computer-aided diagnosis.

### Appendix A.2

As shown in Figure A2, the model incorrectly downstaged a T3 rectal cancer to T2. This highlights the limitations of deep learning algorithms in identifying minor anatomical invasion. The key pathological distinction between T2 and T3 stages is whether the tumor penetrates the muscularis propria and invades the surrounding perirectal fat. However, due to the inherent soft-tissue contrast and spatial resolution limits of CT, minor invasion in early T3 tumors often appears as bowel wall thickening with blurred margins rather than clear fat involvement. These “pseudo-benign” imaging signs contribute to missed radiological diagnoses. In terms of feature extraction, CNNs build high-level semantic representations through successive convolution and pooling operations. This process inevitably causes the loss of high-frequency edge information. Combined with the partial volume effect during imaging, the microscopic invasion features at the lesion margins are smoothed out. Ultimately, the algorithm relies too heavily on the macroscopic morphology of the main lesion and fails to capture the subtle visual evidence of muscularis penetration, leading to downstaging.

### Appendix A.3

Figure A3 displays the changes in the loss function and accuracy during the training process. The training loss decreases gradually with iterations and eventually stabilizes, indicating good model convergence. Also, the accuracies of both the training and validation sets improve steadily and plateau in the later epochs. The trends of the training and validation curves are highly consistent, which shows that the model has good generalization ability without obvious overfitting. The slight fluctuations observed in the validation curve may be related to the inherent heterogeneity and complexity of the medical imaging data.

### Appendix A.4

To evaluate the interpretability of the model predictions, we used the Grad-CAM method to visualize the attention regions of different models (Figure A4). Compared to the ViT and Convnext models, our method shows a more concentrated response in the tumor region. It effectively reduces background noise interference and demonstrates higher consistency with manually annotated ROIs. These results indicate that the proposed regional attention mechanism effectively improves the model’s ability to localize key lesion areas. Based on a preliminary evaluation by medical experts, the high-attention regions of the model match the actual lesion locations well, reflecting reasonable clinical diagnostic logic.

### Appendix A.5

To evaluate the model’s performance on the class-imbalanced dataset, we calculated the precision, recall, and F1-score for each category, along with the macro-average and micro-average metrics (Table A1). The macro-average is the unweighted arithmetic mean across all classes. It treats each class equally regardless of sample size. The micro-average aggregates global true positives, false positives, and false negatives. As shown in the results, the model achieved stable performance across all categories. T3 showed the best performance (F1-score = 0.8163). T4 yielded a lower F1-score (0.6667) due to its smaller sample size, but this result remains reasonable. The macro-average F1-score was 0.7372. This indicates that the model avoided severe bias toward the majority class and achieved a good balance. The micro-average F1-score, which is numerically equivalent to the overall accuracy, was 0.7600. This demonstrates the overall classification reliability of the model.

**Table A1:** Accuracy and macro-average metrics of the ROITransStage multi-classification model.

	Precision	Recall	F1-Score
T1	0.7273	0.8	0.7619
T2	0.6786	0.7308	0.7037
T3	0.8511	0.7843	0.8163
T4	0.6429	0.6923	0.6667
Macro-Avg	0.7249	0.7518	0.7372
Micro-Avg	0.76	0.76	0.76
